# Ecology and Demography of Free-Roaming Domestic Dogs in Rural Villages near Serengeti National Park in Tanzania

**DOI:** 10.1371/journal.pone.0167092

**Published:** 2016-11-28

**Authors:** Anna M. Czupryna, Joel S. Brown, Machunde A. Bigambo, Christopher J. Whelan, Supriya D. Mehta, Rachel M. Santymire, Felix J. Lankester, Lisa J. Faust

**Affiliations:** 1 Department of Biological Sciences, University of Illinois at Chicago, Chicago, Illinois, United States of America; 2 Serengeti Health Initiative, Lincoln Park Zoo, Chicago, Illinois, United States of America; 3 Department of Epidemiology and Biostatistics, University of Illinois at Chicago, Chicago, Illinois, United States of America; 4 Paul G. Allen School for Global Animal Health, Washington State University, Pullman, Washington, United States of America; University of Southern Queensland, AUSTRALIA

## Abstract

Free-roaming dogs (*Canis lupus familiaris*) are of public health and conservation concern because of their potential to transmit diseases, such as rabies, to both people and wildlife. Understanding domestic dog population dynamics and how they could potentially be impacted by interventions, such as rabies vaccination, is vital for such disease control efforts. For four years, we measured demographic data on 2,649 free-roaming domestic dogs in four rural villages in Tanzania: two villages with and two without a rabies vaccination campaign. We examined the effects of body condition, sex, age and village on survivorship and reproduction. Furthermore, we compared sources of mortality among villages. We found that adult dogs (>12mos) had higher survival than puppies in all villages. We observed a male-biased sex ratio across all age classes. Overall survival in one non-vaccination village was lower than in the other three villages, all of which had similar survival probabilities. In all villages, dogs in poor body condition had lower survival than dogs in ideal body condition. Sickness and spotted hyena (*Crocuta crocuta*) predation were the two main causes of dog death. Within vaccination villages, vaccinated dogs had higher survivorship than unvaccinated dogs. Dog population growth, however, was similar in all the villages suggesting village characteristics and ownership practices likely have a greater impact on overall dog population dynamics than vaccination. Free-roaming domestic dogs in rural communities exist in the context of their human owners as well as the surrounding wildlife. Our results did not reveal a clear effect of vaccination programs on domestic dog population dynamics. An investigation of the role of dogs and their care within these communities could provide additional insight for planning and implementing rabies control measures such as mass dog vaccination.

## Introduction

Domestic dogs (*Canis lupus familiaris*) suffer from and can be reservoirs of diseases such as rabies and canine distemper. In Africa, these viruses threaten wildlife such as lions (*Panthera leo)* [1–3], spotted hyenas (*Crocuta crocuta)* [2,4], and African wild dogs (*Lycaon pictus*) [5–7]. Elsewhere they threaten black-footed ferrets (*Mustela nigripes)* [8,9], giant pandas *(Ailuropoda melanoleuca)* [10], Amur tigers *(Panthera tigris altaica)* both in the wild [11] and in captivity [12], chilla foxes (*Lycalopex griseus*) [13], Indian foxes (*Vulpes bengalensis)* [14] and others [9,15–18]. Domestic dogs are also the primary source of rabies in people, with bites from rabid dogs causing more than 95% of human rabies cases worldwide [19–21]. Rabies causes an estimated 55–59,000 human deaths in Africa and Asia [19,22,23], with an estimated 1,500 human deaths annually in Tanzania alone [24,25]. These concerns for public and wildlife health have led to mass domestic dog vaccination programs in many developing countries including Tanzania. Although such programs have been effective in eliminating rabies outbreaks in domestic dogs [25–28], understanding the demography of the targeted domestic dog populations will be key to future success. Such knowledge can influence logistics such as the quantity of vaccines required and the frequency of vaccination campaigns. However, long-term demographic assessments are rarely included in these campaigns. Here, we studied the ecology of village dogs in four rural villages in northern Tanzania over four consecutive years (2010–2013) in order to determine their rates of survival, reproduction, and causes of death.

Even though domestic dogs are the most wide-spread carnivore in the world [29], much of our knowledge about them is limited to breed-specific clinical research, veterinary or behavioral sciences, or broad scale evolutionary studies of phylogeny and origins [30,31]. Studies on the dogs themselves are concentrated in highly controlled, human-mediated contexts [32,33]. However, the population dynamics of free-roaming dogs can be influenced by human, environmental, and wildlife factors [29,34]. Likewise, it is these dogs that can have profound impacts on the humans, wildlife, other domestic animals, and the environment within which they exist.

Defining what exactly a free-roaming domestic dog is can be quite challenging. Most classifications of domestic dogs pertain to the level of dependency on humans for food and shelter [29,31]. With this metric it is relatively easy to classify typical “pet” dogs whose population dynamics are fully dependent on and controlled by humans, or that of feral dogs, which are self-sustaining and mostly independent of human contact. However, there is much confusion about how to classify the dogs “in the middle”, or the populations that are owned but roam, such as many of the rural dog populations in Tanzania and other areas of Africa and Asia where canine rabies remains endemic. Although such dogs can breed freely among themselves as a population, they typically have an owner and receive provisioning at the household. Even within this population, some dogs are ownerless, or “stray”, but do maintain some contact, mainly food-motivated, with humans [31].

Free-roaming dog studies generally have a disease focus and rely on short-term surveys (often one time-point) where owners report dog demographics [34–42]. Important findings include overall male-biased sex ratios ranging from 1.4:1 in Tanzania [39] to 4.9:1 in rural areas of Chile [42]. Possible explanations include lower female survival rates and/or lower life expectancy, as a cost of reproduction or inability to compete with males for food. For example, Kitala et al. [37] reported an average life expectancy of 3.5 years for males and 2.4 years for females in Kenya. Additionally, there may be a preference for male dogs amongst dog owners because of the belief that males are better guard dogs [37,43] and/or selective killing or disposal of female pups [34].

Studies reveal mean life expectancies ranging from 1.1 years in Zimbabwe [36] to 2.5 years in Ecuador [35] and 3.5 years for male dogs in Kenya [37]. These life expectancies of free-roaming dogs are short compared to the median life expectancy of typical companion dogs in the UK, of 12 years [44] or 10 years in Denmark [45]. However, despite these shorter life spans, many free-roaming dog populations are growing. Population growth rates reported in the literature seem relatively high, including 9.0% in Kenya [37], 6.5% in Zimbabwe [36], and 9.0% in Chile [42] and suggest that these populations are rapidly growing.

These studies suggest that in free-roaming dog populations, 1) life expectancies are approximately two years, 2) mortality is higher for females compared to males and 3) many dog populations appear to be growing, despite short life expectancies. However, many studies on free-roaming dogs are short term (≤1 year duration), often with just one survey or sampling period, which rely mostly on owner surveys [36–38,41]. Such snapshots provide valuable insights and observations for developing hypotheses pertaining to general demographic structure. Yet, these snapshots of dog ecology may fail to reflect longer-term trends and seasonal events such as droughts. Furthermore, what is frequently missing in research involving free-roaming domestic dogs is the combination of individual dogs tracked over time and ownership practice information which could provide insight into long-term dog population growth and individual dog survival [29,34].

The objective of our research was to carry out a longitudinal study investigating the demography, ownership practices and body condition of free-roaming domestic dogs in four villages located near the Serengeti National Park. Two of these villages are of particular interest because dogs here are part of an ongoing central-point rabies vaccination program designed to prevent the transmission of rabies and canine distemper into Serengeti National Park [4,46]. The demography of dogs in these two vaccination villages was compared to that of dogs in two non-vaccination villages to evaluate whether this vaccination program impacts dog demography.

We followed the life histories of individually-marked free-roaming dogs over a four-year period measuring survival rates, body condition, and age distributions. We also incorporated household questionnaires to capture demographic events, such as births and deaths, during the interval between visits, and to determine the state (alive or dead) of dogs not present at the household during the visit. Specifically, we aimed to determine: 1) life expectancies of puppies and adult dogs, 2) whether higher female mortality produces a male-biased sex ratio, 3) how body condition influences mortality rates, 4) the principal sources of mortality, 5) how villages differ in dog demography and whether any village effects such as increased survival and population growth can be ascribed to the vaccination programs, and 6) whether within the vaccination villages, vaccinated dogs have lower rates of mortality than unvaccinated dogs.

## Materials and Methods

### Ethics statement

We obtained permission to conduct research in these villages from the District Executive and Veterinary Offices as well as the Village Executive Officer and village council. This research was evaluated and approved by the University of Illinois at Chicago Institutional Animal Care and Use Committee (IACUC) (ACC # 10–042) and the Lincoln Park Zoo IACUC. The questionnaire and research protocol was reviewed and determined exempt by the University of Illinois at Chicago Institutional Review Board (protocol # 2010–0505). Permission to conduct research in Tanzania was obtained from the Tanzania Commission for Science and Technology (COSTECH) (Permit No. 236-ER-2010-1) and Tanzania Wildlife Research Institute (TAWIRI). We obtained consent from each household owner prior to beginning any data collection via a signed consent form at the beginning of the study in 2010, and verbal consent to continue participating in subsequent years. Household owners were free to decline participating at any time.

### Study area

**Fig 1:**
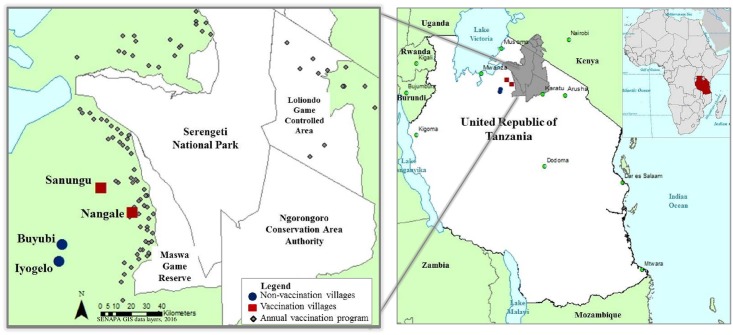
Location of study villages in relation to Serengeti National Park and existing dog rabies vaccination campaign. The location of the four study villages in relation to Serengeti National Park in Tanzania, East Africa is indicated. Nangale and Sanungu villages (red squares) are part of the existing vaccination program while Buyubi and Iyogelo (blue circles) are not part of the vaccination program and therefore “control” villages. Grey lines define the borders of Serengeti National Park and protected areas (Maswa Game Reserve, Ngorongoro Conservation Area Authority, and Loliondo). Grey diamonds indicate the location of the villages where the annual domestic dog vaccination campaign occurred during this research [2,47,48].

Our research took place over four annual field seasons (2010–2013) lasting four months in four villages, two of which were located in Bariadi District (2°48’S; 33°59’E) and two in Maswa District (3°25’S; 34°20’E) of the Simiyu region, west of Serengeti National Park (2°20’S; 34°34’E) in northern Tanzania (Fig 1). Two villages (Sanungu and Nangale) were part of the existing Serengeti Health Initiative domestic dog vaccination program, which vaccinates dogs annually for rabies, canine distemper and parvovirus in villages bordering Serengeti National Park. Two ‘control’ villages (Buyubi and Iyogelo) were outside of this vaccination zone and dogs in these villages were not vaccinated [4]. All of the selected villages were in the same geographical location, and were similar in size, tribal affiliation, and livelihood. People living in these villages were predominantly Sukuma tribe agro-pastoralists growing maize, cotton, and rice while maintaining herds of cattle, goats, and sheep. We collected data annually every August-December 2010–2013. We selected these sampling periods to avoid the heavy rainy season (typically March- May) and the cotton harvest (July-August) during which many household owners would have been unavailable for data collection.

### Household and dog selection

All dogs in this study belonged to specific families. Each village was organized into sub-villages by village authorities prior to the study. We selected households from each of these sub-villages based on the presence of dogs and willingness of the household owner to participate in the study. We included all dogs within a study household in the study, regardless of whether they could be captured and marked. Dogs that were too aggressive to handle, or those that were not present during the household visit (out roaming or herding) were included by collecting basic information from the owner and confirming the dog’s identity with photographs from previous years.

### Dog marking and assessment

Over the course of the study, we marked 1,590 individual dogs in the four study villages. Ear tattoos and photographs were used to mark 877 dogs from 410 households in 2010. These same households were revisited at approximately the same time in 2011 (466 new dogs identified), 2012 (486 new dogs identified), and 2013 (446 new dogs identified) to assess those dogs and mark any new adult dogs or pups. Once captured, we visually assessed dogs for sex, age, and body condition. Body condition (BCS) was scored based on a 1–9 scale (1–2 = poor, 3–4 = fair, 5 = ideal, 6–7 = moderately fat, and 8–9 = obese) [49,50]. Scores were assigned while physically assessing the dog, or, if unable to re-capture, visually assessed from a short distance.

Ages were assigned to dogs based on the age the owner reported at the first visit and birthdates were estimated unless the specific date was known. As owner recall of dog age may potentially be biased, dentition was assessed for the presence of adult canines to confirm that dogs were older than six months. Adult canine teeth typically erupt at around four-five months of age and by five-six months permanent incisors and canines are in place [51]. In addition, we visually assessed dogs to help further confirm ages (although diet and health may influence dentition). To avoid amplifying biases during subsequent visits, we scored the dog’s age as 12 months older than the year before.

### Household questionnaires and village census

During each household visit, a survey (see S1 File) was conducted in Kiswahili or Kisukuma (local tribal dialect) depending on the owner’s preference. Causes of death were recorded as reported by dog owners. In the two vaccination villages (Nangale and Sanungu), owners were asked about participation in the annual dog vaccination campaign. Work in each village was timed to occur directly after the annual vaccination campaign to assist with accurate data collection. Vaccination status of individual dogs was confirmed through vaccination certificates and/or the presence of a vaccination collar. Additionally, all households in the village were visited annually to create a census of the total human and dog population in each of the four study villages (including non-dog owning households).

### Data analysis

Descriptive statistics were analyzed using Microsoft Excel Analyses ToolPak [52]. Data were analyzed using STATA- IC version 12 [53]. We report *p-*values which we considered significant if less than or equal to 0.05 [54–56]. Dogs were censored as “lost to follow-up” from the analysis if the owner declined to participate further, the entire household moved away, or the dog was given away. We compared sex ratios among the four study villages and over the four years using a two way analysis of variance [57]. Dog age was classified into the following age categories: 0–3 months, 4–12 months, 13–24 months, 25–36 months, 37–48 months, 49–60 months, and >60 months. We recorded death dates as the midpoint (the 15^th^) of the owner-reported month and year of death, unless owners reported a specific known date such as “yesterday”. Age at death was calculated by subtracting a dog’s birthdate from the owner-reported date of death. We estimated life expectancy as the mean age at death [58] and assessed these data using a Shapiro-Wilk test for normality [57]. We compared the mean age at death between sexes and amongst villages using a Kruskal-Wallis non-parametric ANOVA [57].

Village, sex, age, BCS, and vaccination status were assessed as predictor variables for annual dog survival over the four year duration of the study using Kaplan-Meier survival estimates and compared for equality with a log-rank test [59]. Variables with significant *p*-values (≤0.05) were included as candidates for the final survival model. Cox proportional hazard models, *h(t*,X) = *h*_*0*_(*t*)*exp* (∑β_i_X_i_) [59,60], were used to compare the hazard ratios *h(t*,X) (hereafter referred to as HR), or mortality rate, at time *t* for dogs with predictor variables (X) including study village, sex, age class, BCS, and vaccination status and to generate adjusted survival curves. To select the reference category, variable categories were sorted alphabetically (village name, sex, vaccination status) and numerically lowest to highest (age class and BCS). We reported HR as an assessment of relative risk of death compared to the reference category. For example, if HR< 1, the relative risk of death for dogs in that category was lower than the reference category and if HR >1, the relative risk was that much higher than the reference [59]. BCS and vaccination status were treated as lag variables, because the data recorded from the previous visit were used as a determinant for present survival. Newly identified dogs from the last study year or puppies of unknown sex were not included in this analysis. This model was assessed for proportionality by estimating scaled Schoenfeld residuals for each variable and testing for nonzero slope [59,61].

Reproductive events reported by dog owners were summarized to obtain mean age at reproduction, litter size, and litter sex ratio. Seasonality was assessed with the Pearson χ^2^ test [54] of the monthly frequency of litters born throughout the year. A Kruskal-Wallis test [54] was used to compare the mean number of puppies produced per litter amongst the villages. We compared the litter sex ratio with a Pearson χ^2^ test.

Dog and human population growth rates were estimated using the annual village-wide census and by estimating the instantaneous rate of increase, *r* (per year), as the natural log of the geometric mean of lambda (λ = N_t+1_/N_t_) 2010–2013 or r = ln(λ_2010–2011*_ λ_2011–2012*_ λ_2012–2013_) [58]. We assessed differences in the total number of dogs recorded as a response variable using generalized linear models with a Poisson distribution [55,56]. We compared candidate models with village (Buyubi, Iyogelo, Nangale, Sanungu), year (2010, 2011, 2012, 2013), and an interaction term (village*year) as predictor variables. We selected the best model using the Akaike information criterion (AIC) [55,56]. We analyzed BCS counts individually across villages, sex, and age classes using a Pearson χ^2^ test [54]. We modeled village, sex, and age class as predictor variables with BCS as the response variable for each year using an analysis of variance (ANOVA) [41].

One of our objectives was to investigate the influence of vaccination on domestic dog population dynamics and compare demographic data between dogs living in vaccinated and unvaccinated villages. However, because grouping study villages into vaccination and control groups may obscure differences among the villages, we first analyzed the four villages separately. We combined them into vaccination and control groups only where there were no differences between the two villages within that group.

## Results

### Study population

In the 420 households surveyed over four years (1,611 surveys collected), dog owners reported that dogs were kept primarily for livestock and household protection (98.7%). Only two households (0.4%) reported using dogs solely for hunting and one household (0.2%) reported keeping dogs only for companionship. Dogs roamed freely and were rarely restrained. Owners fed their dogs a diet consisting primarily of ugali (maize flour paste), potatoes, and occasionally milk. Village leaders, household owners and livestock officers reported very few (if any) stray or un-owned dogs in all villages.

**Fig 2:**
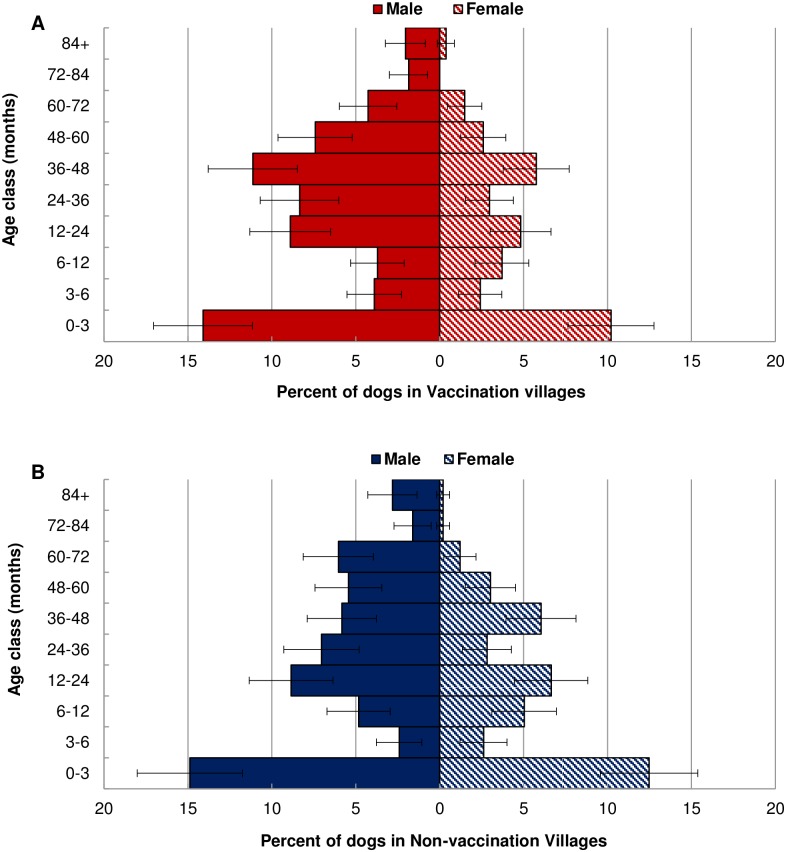
Age and sex distribution of dogs alive in 2013 (*n* = 1,028). Percent of males (solid bars) and females (hashed bars) in each age class (months). Red bars (A) indicate vaccination village dogs and blue bars (B) indicate non-vaccination control village dogs. Error bars indicate 95% confidence intervals.

During the course of the study, 2,649 dogs were observed across 420 households (Table 1). An initial cohort of 1,243 dogs was individually identified, photographed and enrolled into the study in 2010 (Table 1). Between 2011 and 2013, we identified 1,398 new dogs and puppies. During the study, 76 dogs (3%) from 24 households were lost to follow up because either the household moved away (20 households, 60 dogs [2%]) or the head of the household declined to participate further in the study (4 households, 16 dogs [0.6%]). The study population consisted of 1,565 males (60%) and 1,022 females (39%). Sixty-two puppies were not sexed (2%) because the mother was too aggressive to assess the litter or the litter was located in an inaccessible area. In all villages, the sex ratio, which ranged 1.1–2.4:1 (M:F), was male-biased and varied significantly amongst the four villages and across the four years (F_3,12_ = 9.06, *p*<0.01) (Table 1). We report the 2013 age distribution (Fig 2) because at this time point ages were known for most of the adults in the study. Similar to 2010–2012, the age distribution in 2013 was male-biased and heavily skewed toward puppies with most of the total population consisting of puppies 0-3mos old (27%) (Fig 2).

**Table 1:** Numbers of dogs and households enrolled in each village 2010–2013.

	Non-vaccination		Vaccination			
	Buyubi	Iyogelo	Nangale	Sanungu	Total	Mean
Number of study households	114	101	98	107	420	105
Total no. of study dogs	638	676	688	647	2649	662
Mean dogs per household*	5.60	6.69	7.02	6.05	-	6.31
Initial No. of dogs enrolled 2010**	320	307	316	300	1243	311
No. of new dogs enrolled:						
2011	106	122	117	121	466	117
2012	107	122	126	131	486	122
2013	104	119	129	95	447	112
Total no. males enrolled	381	367	437	380	1565	391
Total no. females enrolled	238	302	242	240	1022	256
Total no. unknown sex***	19	7	9	27	62	16
Sex ratio (M:F):						
2010	1.6:1	1.1:1	1.9:1	1.4:1	-	1.5:1
2011	1.9:1	1.3:1	2.3:1	1.7:1	-	1.8:1
2012	2.4:1	1.3:1	2.1:1	1.9:1	-	1.9:1
2013	1.6:1	1.3:1	2.0:1	1.8:1	-	1.7:1

General study household and dog enrollment over the course of the study. Study households were revisited annually in 2011, 2012, and 2013 after initial enrollment in 2010.

*Overall mean number of dogs observed per household throughout the study

**Number of dogs enrolled in study in each year. 2011–2013 numbers represent total number of new dogs and puppies enrolled that year

***Number of puppies we were unable to sex

### Life expectancy

The mean age for the 1,036 dogs alive in 2013 was 28.3 months (median = 20.0 months; range: 1 day-11 years; 95% CI 26.6–29.8 months). Across the entire sample, the mean age at death was 25.8 months (median = 14.2 months; range: 7 days– 13 years; 95% CI 24.3–27.3 months). Mean age at death was not normally distributed (Shapiro-Wilk test, W = 0.81, *p*<0.01) and differed among the villages (Kruskal-Wallis test, H = 31.8, 3 d.f., *P<*0.01). Mean age at death was lower in Iyogelo village (non-vaccination) at 20.7 months (95% CI 18.2–23.2 months) compared to Buyubi, Nangale and Sanungu where the pooled mean age at death was 27.7 months (95% CI 25.9–29.5 months) (Kruskal-Wallis test, H = 4.05, 2 d.f., *P =* 0.13). Mean age at death was slightly lower for females (24.8 months, 95% CI 22.3–27.4 months) but did not differ significantly (Kruskal-Wallis test, H = 0.23, 1 d.f., *P =* 0.63) from that of males (25.1 months, 95% CI 22.8–27.3 months) in all villages except Sanungu village. Here, female mean age at death was 31.9 months (95% CI 22.3–27.4 months) and higher (Kruskal-Wallis test, H = 5.26, 1 d.f., *P =* 0.02) than males whose mean age at death was 26.0 months (95% CI 21.8–30.3 months).

### Survival analysis

**Fig 3:**
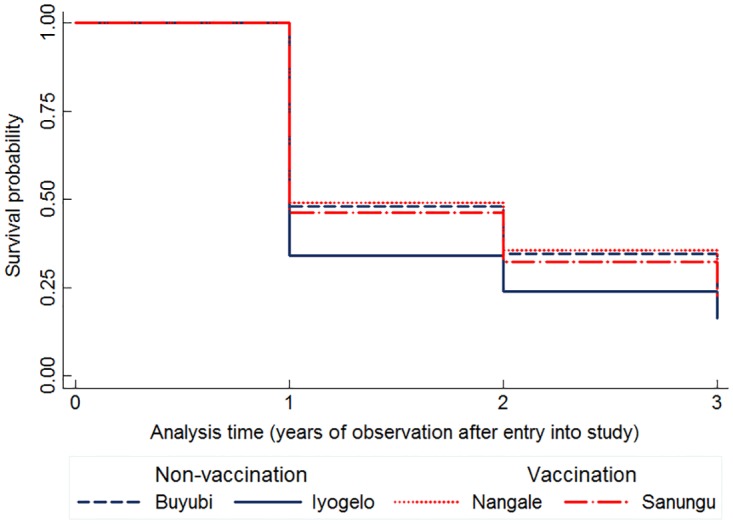
Kaplan-Meier survival estimates indicating the probability of a dog surviving until the next year of observation after it was first encountered. Dogs in Iyogelo village (non-vaccination, indicated by solid blue line) had lower survival probabilities compared to dogs in Buyubi (non-vaccination, blue dashed line), Nangale and Sanungu (vaccination, red dotted line and dash-dot respectively) throughout the study period. The x-axis (analysis time) indicates the year of observation after a dog was enrolled into the study and the y-axis indicates the survival probability.

Of the total number of dogs enrolled 2010–2012, a mean of 28% survived into 2013 with 31% survival in Buyubi, 22% in Iyogelo, 32% in Nangale and 28% in Sanungu. Dog survival differed significantly among the villages (F_3,2636_ = 4.33, *p*<0.001). The Kaplan-Meier survivor estimates revealed that the survival probabilities of dogs in Iyogelo village (non-vaccination) were significantly lower (log-rank χ^2^ = 135, *p* <0.01) than the other three villages, including the other non-vaccination village, Buyubi (Fig 3). When age class, BCS, sex, vaccination status and village were included in the full Cox proportional hazards model, Iyogelo village had a significantly higher probability (24%) of death (HR = 1.24, *p*<0.01) than Buyubi village (reference) (Table 2). Nangale and Sanungu village had 7% and 9% higher risks of death, respectively, than Buyubi but these differences were not significant (Table 2). All age classes had a significantly lower risk of death than the reference category, puppies 0–3 months old (Table 2). The hazard ratios assumed a U-shaped pattern of mortality rates with age, with lower risk of death for adults compared to puppies and senior dogs. The risk of death of dogs 24–36 months old was 62% lower than the 0–3 month old puppies. Probability of death of dogs older than 36 months increased with age while remaining substantially below than that of the puppies (Table 2). Males had a 10% lower probability of death than females (Table 2). There was no effect of pregnancy on female risk of death in the full model (HR = 0.85, *p* = 0.37). This could be an artifact of small sample size as the total number of observably pregnant females at the time of visit was 36 (7% of all females) in 2010, 28 (4%) in 2011, and 28 (3%) in 2012.

**Table 2:** Hazard ratios, standard errors, 95% confidence intervals and *p*-value results of multivariable adjusted Cox proportional hazards model comparing the risk of death across villages, age categories, BCS, sex, reproduction, and vaccination status.

Variable	Category	Hazard Ratio	Std Error	95% CI	*p*-value
Village	Buyubi-control	Reference			
	Iyogelo-control	1.22	0.07	1.09–1.36	<0.01
	Nangale-vaccination	1.08	0.07	0.95–1.22	0.22
	Sanungu-vaccination	1.08	0.07	0.96–1.22	0.19
Age class	0-3mos	Reference			
	3-6mos	0.60	0.04	0.53–0.69	<0.01
	6-12mos	0.49	0.04	0.42–0.56	<0.01
	12-24mos	0.32	0.03	0.26–0.38	<0.01
	24-36mos	0.32	0.04	0.25–0.40	<0.01
	36-48mos	0.42	0.05	0.34–0.52	<0.01
	48-60mos	0.51	0.06	0.40–0.64	<0.01
	60mos+	0.60	0.06	0.49–0.74	<0.01
Body condition score	1–2	Reference			
	3	0.78	0.04	0.70–0.87	<0.01
	4	0.73	0.04	0.64–0.82	<0.01
	5–7	0.73	0.05	0.63–0.83	<0.01
Sex	Female	Reference			
	Male	0.90	0.04	0.83–0.98	0.01
Reproductive state	Not lactating	Reference			
	Lactating	0.93	0.14	0.69–1.25	0.63
Vaccination status	Non-vaccinated	Reference			
	Vaccinated	0.72	0.05	0.63–0.82	<0.01

Hazard ratios (HR) indicate the relative risk of death for specific conditions when all other variables are held constant. Model is simultaneously adjusted for all variables presented.

### Reproduction

**Fig 4:**
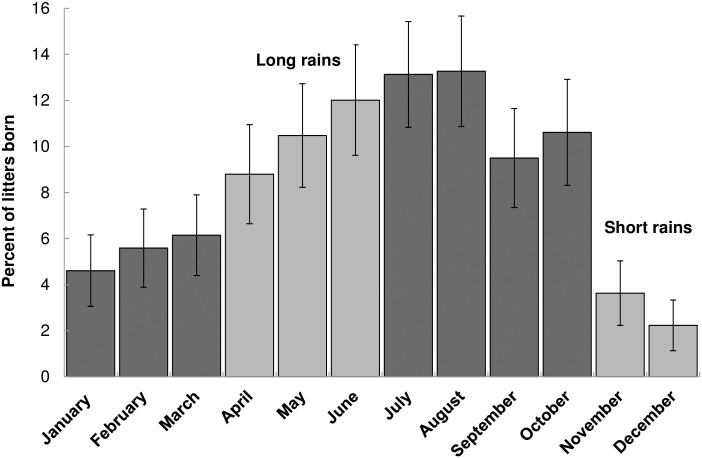
Percentage of litters of puppies born (*n* = 716) throughout the study in each month of the year. Lighter colored bars (March-May and November-December) indicate typical rainy season conditions. Dark colored bars (January-February and June-October) indicate typical dry season. Error bars represent 95% confidence intervals.

Owners reported female dogs whelping as early as six months of age with the oldest female whelping at 9.5 years of age. A total of 391 females (38% of all female dogs of all ages enrolled in the study) gave birth to at least one litter from 2010–2013. Of these, 190 (49%) had one litter within the four year study period, 112 (29%) had two litters, 56 (14%) had three litters, 32 (8%) had four litters and one female (0.3%) had six litters during the study period. Although females whelped throughout the year, a distinct peak in number of litters born occurred in July (n = 94, 13%) and August (n = 95, 13%) (Pearson χ^2^ = 136, *p<*0.01) (Fig 4). A total of 716 litters (3,447 total puppies born during the study period) were reported (151 litters in Buyubi, 202 in Iyogelo, 168 in Nangale, and 195 in Sanungu). Mean litter size, which was 4.9 puppies (range = 1–13) per litter, did not differ significantly among the villages (Kruskal-Wallis test H = 5.2, 3 d.f., p = 0.16). Mean litter sex ratio (male:female) was male-biased (1.2:1) (Pearson χ^2^ = 6.27, *p* = 0.01) and similar in all villages (Kruskal-Wallis test, H = 1.17, 3 d.f., *P* = 0.76). Most litters were born to females 1–2 years old (n = 264, 37%), 2–3 years old (n = 177, 25%), and 3–4 years old (n = 123, 17%).

### Body condition

**Fig 5:**
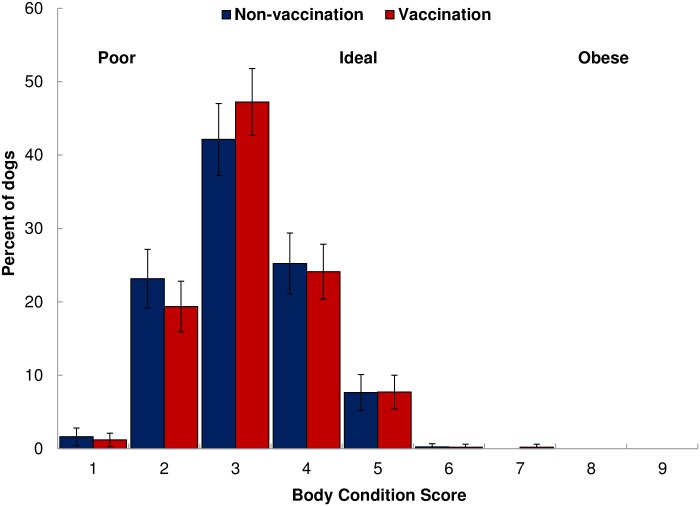
Percentage of body condition scores (BCS) of dogs alive in 2013. Blue bars represent non-vaccination villages (Buyubi and Iyogelo). Red bars represent vaccination villages (Nangale and Sanungu). Body condition was scored as 1–2 = poor; 5 = ideal; 8–9 = obese. We did not observe any obese dogs throughout the study. Error bars indicate 95% confidence intervals.

BCS did not vary significantly between vaccination and control zones (F_1,936_ = 0.53, *p* = 0.47), or amongst the four study villages in 2013 (F_3,934_ = 1.98, *p* = 0.12) and in 2012 (F_3,997_ = 2.38, *p* = 0.07), but did vary among the villages in 2011 (F_3,936_ = 8.0, *p*<0.01) and in 2010 (F_3,877_ = 8.25, *p*<0.01). In 2013, the overall mean BCS was 3.40 ± 0.05 or fair. BCS was right-skewed where most dogs (45%) had a BCS of 3, followed by 4 (25%), and 2 (21%). Only 8% of dogs had a BCS of 5 (ideal) or more and 1% were in a very poor BCS of 1 (Fig 5). BCS of lactating females was 2.56±0.06 and significantly lower (F_7,1400_ = 17.81, *p*<0.01) than non-lactating females which had a mean BCS of 3.31±0.03. Within the villages, BCS did not differ significantly between males and non-lactating females in 2013 (F_4,894_ = 1.63, *p* = 0.17), but did differ between the sexes in 2012 (F_4,950_ = 3.71, *p*<0.01), in 2011 (F_4,897_ = 8.65, *p*<0.01), and in 2010 (F_4,831_ = 6.83, *p*<0.01). Males tended to have higher mean BCS in all villages and years except for in Nangale village in 2010 where the mean male BCS was 3.15±0.07 and mean female BCS was 3.29± 0.10. When including village, sex, and age class as predictor variables for BCS, all variables significantly affected BCS in 2010 (F_11,869_ = 6.29, *p*<0.01) and in 2011(F_11,928_ = 5.53, *p*<0.01). However, village was not a significant predictor in 2012 (*p* = 0.11) and 2013 (*p* = 0.28).

**Fig 6:**
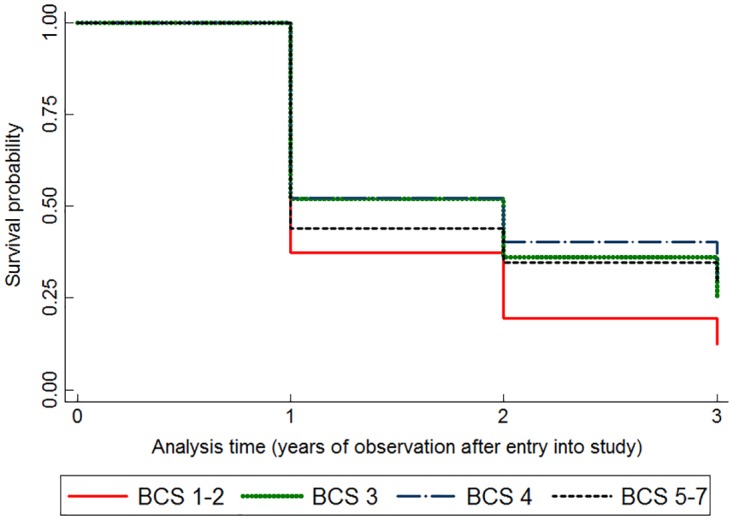
Kaplan-Meier survival estimates for survival probabilities of dogs within different body condition scores (BCS). BCS was scored as 1–2 = poor; 5 = ideal; 8–9 = obese. Dogs with BCS 1–2 (solid red line) had significantly lower survival probabilities compared to all other categories regardless of village, age, sex, or vaccination status.

BCS was associated with survival when included in the full Cox proportional hazards model regardless of village, sex, age, and vaccination status. Body condition impacted mortality, with dogs in better condition having significantly lower risk of death. Compared to dogs with a BCS of 1–2, dogs with a BCS of 3 had a 21% lower risk of death, and dogs with a BCS of 4 and 5 had a 27% lower risk of death (Table 2 and Fig 6).

### Causes of death

**Fig 7:**
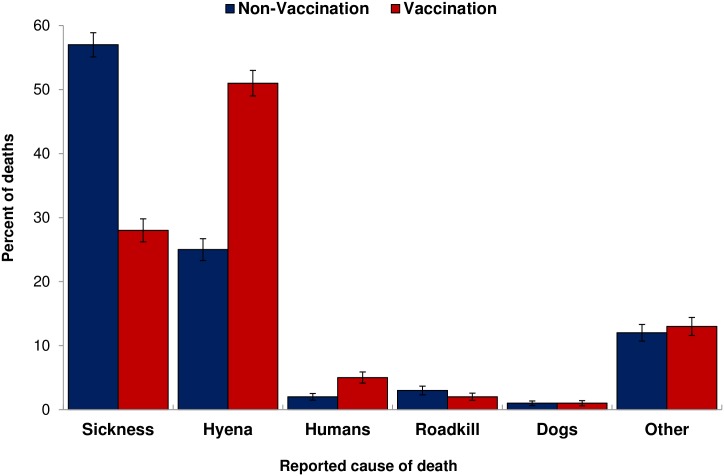
Percentage of owner-reported causes of death of dogs enrolled in the study. Blue bars represent non-vaccination villages (Buyubi and Iyogelo). Red bars represent vaccination villages (Nangale and Sanungu). Error bars represent 95% confidence intervals.

Owners reported 1,253 deaths between 2010 and 2013 and were able to provide a suspected cause for 1,103 of these deaths (88%). Overall, the majority of deaths were due to ‘sickness’ (43% of deaths, 95% CI: 40–46%) and hyenas (38% of deaths, 95% CI: 35–41%). Less frequently reported causes of death included killed by people (3%, 95% CI: 2–4%), road kill (2%, 95% CI: 2–3%), and being mauled by other dogs (1%, 95% CI: 0–1%). Other factors responsible for 13% (95% CI: 11–15%) of deaths included reported leopard predation, snake bites, trampling by cows, failure to nurse, and sudden death. Because the frequencies of causes of death did not differ between the two control villages (F_1,632_ = 0.76, *p* = 0.38) or between the two vaccination villages (F_1,606_ = 0.51, *p* = 0.48), the frequencies were combined, respectively. Frequencies of causes of death differed between vaccination and control villages (F_1,1240_ = 19.81, *p*<0.001) (Fig 7). The majority of the 634 deaths in the two non-vaccination villages were due to sickness (N = 371, 58% of deaths, 95% CI: 54–62%) and hyena predation (N = 163, 25% of deaths, 95% CI: 21–28%). In contrast, the majority of the 608 deaths in the vaccination villages were due to reported hyena predation (N = 313, 51% of deaths, 95% CI: 48–55%) followed by sickness (N = 168, 27% of deaths, 95% CI: 24–31%). Typically, owners reported hyenas killing dogs at the household when dogs attempted to attack and chase away a hyena that was likely hunting for livestock.

### Population growth

**Fig 8:**
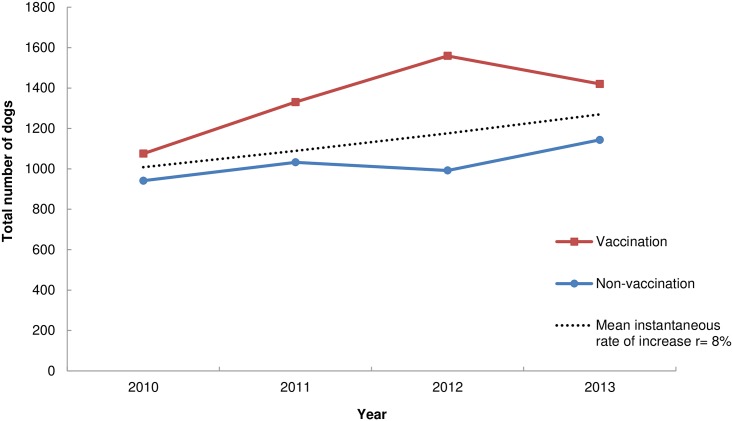
Total number of dogs recorded each year of the study in each village census. The blue line represents the non-vaccination villages (Buyubi and Iyogelo) and the red line represents the vaccination villages (Nangale and Sanungu). The trendline indicates the mean predicted instantaneous rate of increase (r = 0.08 per year) between 2010 and 2013 in all villages.

Between 2010 and2013, the village census revealed an overall increase in the total number of dogs. The rate of increase varied from year to year (Table 3). The GLM analysis revealed that the model with the interaction term village*year had a higher AIC value (12.34). We therefore selected the best model (AIC 12.33) which included only the predictor variables village and year. Total number of dogs varied amongst the villages and across the years (Fig 8) (GLM village, year F = 15.1, 13.9; df = 3, 1; *p*<0.01). The differences in the intercepts among the villages revealed that the number of dogs vary, but that the overall rate of growth (exponent of year coefficient, *e*^0.077^ = 1.08) was 8% and similar across the villages (Table 4). We also observed the human population increasing in all villages during the study period (GLM village, year F = 97.4, 12.6; df = 3, 1; *p*<0.01). Overall human population growth from 2010 to 2013 was 4%. The human: dog ratio ranged from 6:1 to 9:1 (Table 3). In 2013, the human: dog ratio was 7.06:1 in Buyubi, 7.03:1 in Iyogelo, 7.61:1 in Sanungu, and 7.02:1 in Nangale.

**Table 3:** Study cohort, village dog and human population census totals and estimated growth rates 2010–2013.

	Non-vaccination		Vaccination		
	Buyubi	Iyogelo	Nangale	Sanungu	Overall total
Number of dogs (Study cohort %*)					
2010	520 (62%)	421 (73%)	569 (56%)	506 (59%)	2,016
2011	537 (53%)	495 (53%)	710 (43%)	620 (49%)	2,362
2012	512 (52%)	480 (50%)	835 (38%)	724 (41%)	2,551
2013	658 (40%)	485 (49%)	758 (40%)	662 (37%)	2,563
Dog population growth (per year)					
r = ln(λ _2010–2013_)**	0.08	0.05	0.10	0.09	0.08
Number of households					
2010	555	494	581	488	2,118
2011	588	502	672	563	2,325
2012	588	472	644	568	2,272
2013	599	514	689	576	2,378
Number of people					
2010	4,285	3,077	5,148	3,760	16,270
2011	4,694	3,205	5,774	4,349	18,022
2012	4,622	3,109	5,401	4,443	17,575
2013	4,646	3,409	5,766	4,647	18,468
Human population growth (per year)					
r = ln(λ _2010–2013_)**	0.03	0.03	0.04	0.07	0.04
Human: Dog ratio					
2010	8.24	7.31	9.05	7.43	
2011	8.74	6.47	8.13	7.01	
2012	9.03	6.48	6.47	6.14	
2013	7.06	7.03	7.61	7.02	

Village census data was collected by visiting each household within each study village annually during the study period.

*Percent of total village dogs enrolled in this study

**r = ln(geometric mean of λ_2010–2011_, λ_2011–2012_, λ_2012–2013_)

**Table 4:** Summary statistics of GLM best model assessing effects of village and year on the total number of dogs.

Variable	Coef.	Std. Error	z-value	*P-*value	95% CI		d.f.	Log Likelihood	AIC
Intercept-Buyubi	-149.1	18.5	-5.39	<0.01	-185.4	-112.8	11	-93.61	12.326
Iyogelo village	-0.169	0.031	9.01	<0.01	-0.23	-0.107			
Nangale village	0.254	0.028	4.14	<0.01	0.199	0.31			
Sanungu village	0.12	0.029	8.39	<0.01	0.063	0.177			
Year	0.077	0.009	-8.05	<0.01	0.059	0.095			

### Status and effects of vaccination

A central-point vaccination was conducted annually in Nangale and Sanungu villages. Not all dogs in these villages participated in vaccination and of those that did, few were consistently vaccinated each of the four years of the study. For example, in our cohort, only nine (1%) dogs were vaccinated each of the four study years. In Nangale, vaccination coverage of enrolled dogs was 32% in 2010, 29% in 2011, 22% in 2012, and 24% in 2013. In Sanungu, vaccination coverage of enrolled dogs was 34% in 2010, 27% in 2011, 19% in 2012, and 21% in 2013.

There were no differences (Pearson χ^2^ = 2.43, *p* = 0.12) in dog survival between vaccination and control villages when comparing crude mortality rates (total number dog deaths/total number dogs enrolled 2010–2012) between vaccination (56%) and non-vaccination (59%). However, there were differences (Pearson χ^2^ = 4.70, *p* = 0.03) in the crude mortality rates between the two control villages, Buyubi (56%) and Iyogelo (63%), and we therefore analyzed village survival probabilities separately rather than combining into vaccination and control groups. When individual vaccination status was included as a variable in the full Cox proportional hazards model, vaccination in the previous year had a protective effect and afforded dogs 30% decreased risk of death compared to unvaccinated dogs (Table 2).

## Discussion

Although domestic dogs have been the focus of many epidemiological studies focusing on rabies vaccination, few have assessed how individual dog metrics, such as body condition, can influence survival. Our longitudinal study demonstrates that the free-roaming domestic dogs in rural Tanzanian villages share many of the same demographic characteristics of other free-roaming dog populations and provides insight about the factors that may limit dog population growth.

Similar to other free-roaming dog studies in Chile [17,42], South Africa [62], India [63], Kenya [37], Bangladesh [64], Bali [62], Mexico [38], Bolivia [65], Bhutan [66] and Thailand [67], sex ratio was male-biased. This is likely a combination of male-biased birth sex ratio, and lower female survival, regardless of village or body condition. Female life expectancy, however, did not differ from that of male dogs, which was also observed in Chile [42] and in Zimbabwe [36]. Decreased female survivorship could result from preferential treatment by owners for male dogs (Czupryna, personal observation), competition for food between the sexes, and costs of reproduction (note these are not mutually exclusive). Preference for male dogs has been reported in some studies [17,37,42,62], because of the belief that male dogs make better guard dogs [29]. A similar study of free-roaming dogs in South Africa, however, reported male-biased sex ratio, even though females did not have higher mortality during one of the study years [68] and a study of dogs in an urban center of central Tanzania found no differences in survival rates between sexes [69]. Lactating females had lower BCS than non-lactating females, suggesting that reproduction has costs. Although costs of reproduction may influence female survival, significant effects of being pregnant on the risk of death in females were not observed when controlling for village, age, and body condition. A closer examination of ownership practices and perceptions could offer more insight into the differences in survivorship between the sexes.

Despite lower survival probabilities, 38% of females gave birth to at least one litter of puppies during the study. Total number of litters and litter size were similar amongst the villages, suggesting that the presence of a vaccination campaign does not influence reproductive rates. Similar to studies in Zimbabwe [36] and India [70,71], there were pronounced seasonal effects on reproduction. Most litters were born between June and August, which coincides with the beginning of dry season (June- November) after the seasonal rains (March-May) in the study area. Domestic dog gestation length is 62–64 days [72], suggesting that dogs are more likely to become pregnant (and later successfully whelp) toward the end of the rainy season during periods of high resource availability. This is consistent with a study from Mexico that found lower pregnancy rates during the warm- dry season and lower pregnancy rates among underweight females[73]. Totton et al. [74] reported higher prevalence of pregnancies during late monsoon season in Jodhpur, India, but suggested that this may be a function of better sperm quality during the cooler monsoon season. Regardless, this suggests that although these are human-mediated domestic animals, they still are subject to environmental pressures.

Mean BCS was fair and similar across all villages. These findings are similar to other studies which report that free-roaming dogs are typically in less than ideal body condition [34,75], but not necessarily poor body condition, as was also noted by Morters et al. [62] While all study dogs were owned and received provisioning at their household at least once daily, which is consistent with free-roaming populations in South Africa and Bali that were mostly fed daily as well [62], these are working dogs (livestock and household protection) with high caloric needs, with often less than ideal food sources (primarily maize flour based diets with little meat or protein sources). Consequently, it was not surprising, that no fat or obese dogs were observed in this study. As expected, body condition influenced survival in all villages regardless of vaccination status, with dogs in poor body condition having a higher risk of death. Because all dogs enrolled in the study were owned and associated with specific households, dog ownership practices (such as type and frequency of food provided) are likely to have an impact on body condition. These data suggest that this population of dogs is influenced by resource availability and that the human owners providing those resources mediate dog survival and population growth. Additional analyses into other ownership practices that may influence dog survival, including the use of parasite preventatives (such as flea and tick spray) and total number of dogs owned per household, could provide more insight about factors influencing domestic dog ecology and demography in these villages.

The survival analyses revealed that survivorship differed significantly among villages, irrespective of vaccination status or zone. Risk of death in Iyogelo (non-vaccination), was greater than that in Buyubi (also non-vaccination), as well as the two vaccination villages, Nangale and Sanungu. Household survey data and interviews with local veterinary officials suggested that Iyogelo experienced a rabies and/or canine distemper outbreak in 2010 and 2011(Czupryna, unpublished data). Causes of death were not confirmed, but in 2011 in Iyogelo, 15 dogs (11% of that village’s deaths for that year) in the study were reported to have died after exhibiting rabies-like symptoms (aggression, abnormal behavior, increased salivation, etc.). Iyogelo village leaders also reported at least one human rabies death, resulting from the bite of a rabid dog, which supports the theory that an outbreak may have occurred in Iyogelo. Both Iyogelo and Buyubi had unvaccinated and susceptible dog populations, and therefore were equally susceptible to a rabies outbreak, but village and veterinary authorities only reported a rabies outbreak in Iyogelo (Czupryna, personal communication).

The suspected outbreak in Iyogelo did not appear to affect survival probability in Buyubi village, located some 20km away. Throughout the study we did not observe any movement of study dogs or families between any of the study villages. Suspected rabid dogs are dealt with quickly in rural Tanzania due to the high incidence of human deaths due to rabies [23,25], and although a rabid dog could potentially travel that distance [29,76,77], it would likely be killed in transition [24].

We found that dog survival in Buyubi village (non-vaccination) was similar to the vaccination villages. Perhaps, in the absence of a disease outbreak, vaccination only plays a small role in dog survival. Free-roaming dogs interact with and are part of a very diverse and dynamic environment and as such they may die as a result of factors both human and natural. Acosta-Jamett et al. found that although only 28% of dogs were vaccinated against canine distemper (rabies was not mentioned in this study), the mortality rate of adult dogs was 0.2 or less and the main two causes of death were human activities (41%) followed by disease (35%) [42]. Conan et al. found that rabies vaccination only reduced mortality rates in unvaccinated puppies and argued that this was highly unlikely due to reduced risk of succumbing to rabies [68].

One of the major gaps in free-roaming dog research is an examination of demographic processes without human intervention strategies such as vaccination and/or sterilization. Similar dog survival in Buyubi village and the two vaccination villages may be the norm, a random coincidence, or it could suggest that vaccination itself does not influence survival in the absence of a disease outbreak.

Our study provides evidence that vaccination increases dog survivorship, when considering individual dog vaccination status. Ideally, the impacts of vaccination would be evaluated in a case-controlled study where a completely naïve, or unvaccinated, population of dogs within the same village and household is divided into a vaccination and control group. This observational study lacks this ideal comparison because vaccinated dogs were compared to non-vaccinated dogs living in both the vaccination and non-vaccination villages. We chose this study design to capture village-wide, or population-wide, trends to reflect the current vaccination program activities. Dogs that were both vaccinated and not vaccinated were enrolled to study the village population as a whole and this method was chosen to avoid selection bias by dog owners, who may prefer to bring dogs that are more easily restrained or specific “favorite” dogs to the vaccination point.

Although ideally vaccination programs strive to vaccinate as many susceptible dogs as possible, it is neither logistically feasible nor necessary to do so to control rabies because of the herd immunity that results from vaccinating a certain proportion of the population [78]. For rabies, 70% vaccination coverage has been recommended as this critical threshold [22,78], but lower annual coverage may be sufficient to prevent outbreaks [24,62,68]. In the two vaccination villages, the overall village-wide vaccination coverages derived from the village census and vaccination log book ranged from 30%-80%. The vaccination coverage of individual dogs enrolled in the study, however, was lower (19–34%). Despite this finding, there were no cases of rabies reported in these villages during the study period.

Although survivorship was somewhat related to the vaccination program, causes of death differed between vaccination and non-vaccination villages. In the non-vaccination villages, 57% of deaths were from illness and 25% of deaths were from predation by spotted hyenas. In the vaccination villages, in contrast, the near inverse was true, with most deaths (51%) from reported hyena predation and only 28% of deaths from sickness. This either suggests that vaccination may offer some protection from risk of death due to sickness, or that predation from hyenas is greater in the vaccination villages. Even in Buyubi (non-vaccination), where dog survival did not differ from the vaccination villages, 48% of dog deaths were due to sickness compared to only 24% in Nangale and 31% in Sanungu. Preventing disease through vaccination, as in these villages, however, is not likely to lead to an increase in the overall dog population.

Alternatively, because the existing dog rabies vaccination program targets villages within 10 kilometers of Serengeti National Park, Nangale and Sanungu villages may potentially have a higher abundance of hyena activity. The two non-vaccination villages, however, are located approximately 30 kilometers from Maswa Game Reserve, a hunting concession, which borders southern Serengeti National Park and is also reported to have high densities of carnivores (Czupryna, personal communication with district livestock officer). Current wildlife abundance information for Maswa Game Reserve is not available. Although a 1999 study [79] found a lower number of hyenas responding to calls in Maswa Game Reserve compared to the Serengeti plains, the author mentions that these differences could be a result of different habitat and vegetation type as well as a heightened hyena weariness and consequent call station avoidance because of ongoing hunting activities [79]. It is possible that reported hyena predation events are biased either because dog owners expected compensation or perhaps because they were unwilling to reveal the true fates of dogs. We do not believe that either of these was the case because owners were not shy about revealing they killed dogs for behavioral reasons or that they used dogs for hunting. Incorporating more frequent visits, serology, necropsies, wildlife scat analysis and more careful tracking to verify fates of dogs could provide additional insight.

Hyena presence was confirmed in all the villages using camera traps, and household surveys reported hyena sightings and hyena predation of other domestic animals such as cows and goats in addition to dogs. In all villages, most household owners reported seeing hyenas every day, suggesting that hyena predation risk is similar across the villages. Regardless of location, reported hyena predation was responsible for a significant proportion of dog deaths and suggests that predation by wild carnivores is compensatory to disease, rather than additive.

The most common reasons for people killing dogs was for exhibiting inappropriate behaviors such as stealing eggs, killing chickens, being overly aggressive and for public health and safety when a dog exhibited rabies symptoms. Similarly, dog owners in rural Chile reportedly killed dogs for inappropriate behaviors toward livestock, but unlike our study, also killed puppies for population control [17]. No dogs in this study were killed for population control measures based on owner reports. On the contrary, dog owners in all villages reported a high need for dogs for household and livestock protection stating they needed to replace dogs that had died. This suggests that unlike areas where overpopulation of feral dogs has been a concern, dog population growth in these rural villages could be the result of increasing human demand as the human population continues to grow. These findings support results of other studies reporting that humans play a significant role in dog population size and movement [62,75].

We found that although the total number of dogs fluctuated, overall the dog population increased by 8% in all villages from 2010–2013 (Fig 8). Previous dog studies have reported similar annual growth rates in Kenya [37] and in rural villages in Chile [42]. Dog population numbers likely fluctuate over short time frames but overall remain constant based on human demand. Morters et al. [62], for example, found declining population size at one site and constant population size in another site over the course of a three-year study in South Africa. Based on village censuses, the human: dog ratio fluctuated slightly year to year in each village, but remained similar, suggesting that an increase in the dog population is likely the result of demand in the growing villages and not merely from vaccination. Village leaders and household owners reported very few, if any, stray or feral dogs in the villages, stating that if a dog was thought to be unowned, it would quickly be adopted by a household as demand for dogs was high. Other studies [26,62,80], also suggest that the majority of free-roaming dogs in rural African villages are owned. Ownership practices and human demand, therefore, likely heavily influence dog population dynamics and growth.

## Conclusion

Our study illustrates that free-roaming domestic dog populations are part of a dynamic environment and affected not only by human interventions such as vaccination and ownership practices, but by environmental conditions as well. While our data suggest that vaccination protects against death from disease, other factors, such as wildlife predation, also play a role in dog survival. In particular, predation by wild carnivores may be compensatory to disease-caused death in these populations. This research has important applications for the planning and implementation of domestic dog rabies vaccination programs. First, vaccination alone does not appear to impact dog population dynamics, suggesting that decreased risk of disease will not necessarily result in rapid population growth (compared to un-vaccinated areas) and require population management interventions such as sterilization campaigns. Regardless of vaccination, population turnover is high, and any deaths, including those due to wildlife predation, will decrease overall vaccination coverage and pose challenges for maintaining the critical threshold levels to prevent rabies outbreaks. Finally, the large proportion of dog deaths due to wildlife predation in conjunction with reported wildlife sighting at households suggest a potentially high risk of disease transmission between domestic animals and wildlife. This further illustrates the importance of gaining a better understanding of the ecology of free-roaming domestic dogs not only for public health concerns, but also as extensions of human impacts on the environment.

## Supporting Information

S1 File Household questionnaire. Household questionnaire used to collect dog demography and ownership practices data.
(PDF)
