# Supplementary material for: Ecology and Demography of Free-Roaming Domestic Dogs in Rural Villages near Serengeti National Park in Tanzania
Source: PLoS One. 2016 Nov 28;11(11):e0167092. doi: 10.1371/journal.pone.0167092 (PMC5125679; doi:10.1371/journal.pone.0167092)
Supplement: S1 File — Household questionnaire used to collect dog demography and ownership practices data. (PDF) [file pone.0167092.s001.pdf]

HHID___________________

DODOSO LA UCHUNGUZI WA KICHAA CHA MBWA

1.Date………..............2.Village Buyubi Iyogelo Nangale Sanungu 5.GPS……………..……..

3. Subvillage………………………….. 4. District: Maswa Bariadi 5.GPS……………..…..…

6.Interviewer…………………7.Head of hh………………..………8.Subvillage leader………………………………..

9.Tribe ………………10.Religion…………………specify…………………………11c. Language: Swahili Sukuma

11.House type: mud/grass mud/iron brick/grass brick/iron other:………………… 11b. Choo? Yes No

12.Total no. people in household……………..No. Children (<18yrs)………… 11d. HHscore: 1 2 3

13.Highest level of education: none primary secondary technical university

Maelezo ya mbwa, chanjo na utoaji damu:

14.Total no. dogs in household…….. Dogs less than 6 mths ……. Dogs above 6 mths ………Total Vacc'd……

15.Total no. cats in household……... Cats less than 6 mths………. Cats above 6 mths………...Total Vacc'd…...

Table A: Information on Household's Current Dogs

Name of dog	ID#	Sex	Age mo owner	Age mo interv.	Vacc (y/n)	Vacc ever	Vacc card	Vacc mark	Where got dog?	Time owned

Table B: Information on Household's Current Cats

Name of cat	ID#	Sex	Age (mos) from owner	Age (mos) int	Cat vacc? (Y/ N)	Where did you get this Cat?	How long owned?

16a.Dogs vaccinated? Yes No Control zone

16b. If not vacc'd, why? Too busy out of town boys not available did not hear other:………………………..

16c. In vacc villages, why do you vaccinate your dogs?...

17a. Has there been any problem with your dog/cat after vaccination? Yes/ No.

b. If yes describe……………………………………………………………………………………...

18.What is the role of your dog(s) (circle): companion protection hunting herding Other:………………..

19.If you use your dog for protection, protection from: people wildlife other:……………………….

20.What does your dog bark at? People other dogs wild animals other:…………………………

21.What is the role of your cat(s) (circle): companion rodent control Other:……………………

HHID____________________

22a. Ideally, how many **dogs** would you want? (circle): A.1-2 B. 3-4 C. 5-6 D. 7-8 E. 9+

b.Why? home/livestock security I like dogs Other:……………

23a. Do you think there are problems with having many dogs in your **household**? (circle) Yes No

b. If yes, why? Food shortage fighting other:………………………

24a.Do you think there have been changes in the village dog population in the last two years? (circle) Yes No

b.If the answer is yes, what type of change? (circle) Increase Decrease

25a.Do you think the change in dog population is causing any problem? (circle) Yes No

b.If yes, why? ……..……………………………………………………………………………..

c.If you think dog population changes are a problem in this village, what measures should be taken to mitigate the problem?………………………………………………………………………………………….……

d.If you think the dog population changes are **not** a problem in this village, why do you think so?

………………………………………………………………………………………………………

26.Have you used any measures to control number of dogs you have in the last 2 years? (circle) Yes No

Table C: Methods of Population Control (Ask owners what methods of population control they have used in the past, are willing to consider if population growth is undesirable for both dogs and cats).

Control Method		Used in past		Using currently		Willing to consider using	
		Yes	No	Yes	No	Yes	No
Giving away/selling	Pups						
	Adult dogs						
Killing	Pups						
Confining/tying up	Males						
	Females						
Surgical Sterilization	Males						
	Females						
Contraceptive Injection	Males						
	Females						
OTHER:							

26e.If no to killing pups, ask: why not kill pups to reduce the number of unwanted pups? (circle)

People need dogs It is not good to kill I like puppies Other:………………………………………………..

27a. Have you acquired any dogs/puppies since the last questionnaire day? (circle) Yes No

b. How many? .... If the answer is yes, verify dogs are listed in table A.

28a.Have any dogs or puppies died since the last questionnaire day? (circle) Yes No

b.How many? .... If the answer is yes, fill in Table D on next page….

HHID___________________

Table D: Mortality/ Disappearance/Sick Data of Previously Recorded Dogs

Name of Dog	ID#	Sex	Death age(mos)	Month died/ missing	Cause of death	If died, what clinical signs?

29a.Is there any dog which fell sick in the last 12 months? (circle) Yes No

b.If yes, which dog and describe:……………………………..

c.What did you do with the sick dog? Nothing Used local herbs treated by livestock officer killed dog Other

d.Did dog change behavior? Yes No If dog changed behavior describe the behavior change:

……………………………………………………………...

e.Do you think dog which fell sick had rabies? (circle) Yes No

If yes, why? ....

f.Was the dog bitten by another animal? (circle) Yes No

If yes, which animal bit the sick dog …………..…………………………….

g.If it was a dog, indicate the name of the dog, if known………………………………………………………

And indicate the name of the owner, if known……………………………………………………………….

What happened to the animal which bit your dog? Describe:…………………………………………………..

30a.Where does your dog sleep? Inside home inside boma (but outside house) Outside boma gate

Other?___________________

30b.How often does your dog sleep in same room with children/family? Never 2-3 times weekly everyday

30c.Does your dog ever clean your child (by licking?)? Yes No

30d.How many days a week does your dog eat feces? never 1- 2 3- 4 5-6 everyday

30e.How many times in an average week do you feed your **dog**? (circle):

Never 1-2 3-4 5-6 everyday

30f.Does this change at different times in the year? Yes No If yes, How? ………………………………

30g.Who feeds/takes the dog?.....................................Age………………Sex: male female

30h. Do you spray your dog for fleas/ticks? Yes No If yes, what do you use:…………………………………

30i. Do you bathe your dog? Yes No How often?.........................With what?.............................

HHID____________________

Table E: Dietary Info (Ask what do you feed your dog? check all that apply)

	**Dog eats:**	**Frequency**			**Other?**
**Food**	**yes**	**monthly**	**weekly**	**everyday**	
ugali					
rice					
millet					
potatoes					
chicken					
beef					
goat					
dagaa					
milk					
egg					
rats					
Fruit(specify)					
Veggies (specify)					

31e.Does your dog ever kill its own food? Yes No If yes, what does it kill?..

32a. Do you own livestock? Yes No If yes, how many livestock do you have? Fill in table below

Table F: Livestock Data

Cattle		Goats		Sheep		Donkeys		Chicken	
>6mos	<6mos	>6mos	<6mos	>6mos	<6mos	>6mos	<6mos	>6mos	<6mos

33.Has any female dog reproduced this year? Yes No

Table H: Female Dog Reproduction Data (Ask for each adult female in Table A)

Name of female	ID number	No. of litters in lifetime?	No. litters this yr?	Month(s) whelped	Total Pups

Table I: Female Dog Reproduction – Litter Details

Mother ID #	Litter month	# pups born		#living in house		# pups died/ disappeared		# pups given away	
		F	M	F	M	F	M	F	M

33d.If pups died, what was the cause of death? ……………………………………………………………………

Were pups killed by owner; if yes, why? ....

If pups given away, ask why? ....

Do you prefer to keep one sex over the other? (circle) Yes No Female Male

If yes, why? ....…...

34.Have you seen non-domestic animals in the village in past year? Yes No Please check table below:

**Species**	**Yes**	**How often do you see these?** **Monthly**	**Weekly**	**Everyday**	**Other**
Lion					
Hyena					
Leopard					
Jackal					
Wildcat					
Mongoose					
Bat eared fox					
Wildebeest					
Impala					
Buffalo					
Porcupine					
Dik dik					
Springhare					
Baboon					
Vervets					
Snake					

34a. How many times has livestock been killed by wildlife since last visit?...

b. What animal killed your livestock? Hyena Leopard Jackal Other:……………….

c. How many livestock has been killed? Cows……….. Goats……… Sheep……….

MAELEZO YA KICHAA CHA MBWA

35.Have you heard of any rabies incidence for the past 2 yrs in the village? Yes No

Table L: Rabies Incidence

Species ya mnyama	Date of incident	Some description of incident if possible

36.Have you heard of any rabies incidence in humans in the past 5 yrs in the village? Yes No

Table M: Human Rabies Incidence

Jina	Umri	Jinsi	Date	Clinical signs	Date of bite	Species biting	Died/ Recovered	If died, indicate date

37.Is there anybody in the household who was ever bitten by an animal? Ndiyo Hapana

HHID____________________

Table N: Human Bite Incidence

Jina	Mnyama aliyemng'ata	Tarehe aliyong'atwa	Clinical signs	Alipona/alikufa

38a. Do you know what rabies is? Yes No

b. If yes, please describe…………………………………………………………………………………..

39a. When was the last time you *heard* of a dog rabies case? ………………………………………………….

b. A human case?..

40a. Have you ever actually personally *seen* a dog rabies case? Yes No

b. If yes, when and describe………………………………………………………………………………

c. A human case? Yes No

d. If yes, when and describe…………………………………………………………………………………

41a. Do you feel your family is at risk for rabies? Yes No

b. Do you feel your dog is at risk for rabies? Yes No

c. If yes, why? …………………………………………………………………………………………………

d. Do you feel your dog is at risk for getting sick with other diseases? Yes No

e. If yes, describe……………………………………………………………………………………………

42a. If a dog has rabies in this village, what happens to it?...

b. If *your* dog has rabies, what do you do with it?...

43a. control villages: Are you interested in vaccinating your dog for rabies? Yes No

a. vacc villages: Are you interested in continuing dog vaccinations? Yes No

b. Would you be willing to pay for dog vaccinations? Yes No

c. If yes, how much?...

ASANTE SANA!
